# Novel Computational Approach to Predict Off-Target Interactions for Small Molecules

**DOI:** 10.3389/fdata.2019.00025

**Published:** 2019-07-17

**Authors:** Mohan S. Rao, Rishi Gupta, Michael J. Liguori, Mufeng Hu, Xin Huang, Srinivasa R. Mantena, Scott W. Mittelstadt, Eric A. G. Blomme, Terry R. Van Vleet

**Affiliations:** ^1^Global Preclinical Safety, Abbvie, North Chicago, IL, United States; ^2^Information Research, Abbvie, North Chicago, IL, United States; ^3^Discovery and Early Pipeline Statistics, Abbvie, North Chicago, IL, United States

**Keywords:** Off-targets, machine learning, toxicology, pocket search, gene expression, secondary pharmacology

## Abstract

Most small molecule drugs interact with unintended, often unknown, biological targets and these off-target interactions may lead to both preclinical and clinical toxic events. Undesired off-target interactions are often not detected using current drug discovery assays, such as experimental polypharmacological screens. Thus, improvement in the early identification of off-target interactions represents an opportunity to reduce safety-related attrition rates during preclinical and clinical development. In order to better identify potential off-target interactions that could be linked to predictable safety issues, a novel computational approach to predict safety-relevant interactions currently not covered was designed and evaluated. These analyses, termed Off-Target Safety Assessment (OTSA), cover more than 7,000 targets (~35% of the proteome) and > 2,46,704 preclinical and clinical alerts (as of January 20, 2019). The approach described herein exploits a highly curated training set of >1 million compounds (tracking >20 million compound-structure activity relationship/SAR data points) with known *in vitro* activities derived from patents, journals, and publicly available databases. This computational process was used to predict both the primary and secondary pharmacological activities for a selection of 857 diverse small molecule drugs for which extensive secondary pharmacology data are readily available (456 discontinued and 401 FDA approved). The OTSA process predicted a total of 7,990 interactions for these 857 molecules. Of these, 3,923 and 4,067 possible high-scoring interactions were predicted for the discontinued and approved drugs, respectively, translating to an average of 9.3 interactions per drug. The OTSA process correctly identified the known pharmacological targets for >70% of these drugs, but also predicted a significant number of off-targets that may provide additional insight into observed *in vivo* effects. About 51.5% (2,025) and 22% (900) of these predicted high-scoring interactions have not previously been reported for the discontinued and approved drugs, respectively, and these may have a potential for repurposing efforts. Moreover, for both drug categories, higher promiscuity was observed for compounds with a MW range of 300 to 500, TPSA of ~200, and clogP ≥7. This computation also revealed significantly lower promiscuity (i.e., number of confirmed off-targets) for compounds with MW > 700 and MW<200 for both categories. In addition, 15 internal small molecules with known off-target interactions were evaluated. For these compounds, the OTSA framework not only captured about 56.8% of *in vitro* confirmed off-target interactions, but also identified the right pharmacological targets for 14 compounds as one of the top scoring targets. In conclusion, the OTSA process demonstrates good predictive performance characteristics and represents an additional tool with utility during the lead optimization stage of the drug discovery process. Additionally, the computed physiochemical properties such as clogP (i.e., lipophilicity), molecular weight, pKa and logS (i.e., solubility) were found to be statistically different between the approved and discontinued drugs, but the internal compounds were close to the approved drugs space in most part.

## Introduction

The drug discovery process for small molecules typically starts with large screening campaigns using institutional or commercial compound collections in order to identify chemical matter for lead optimization efforts (Drews, 2000; Bleicher et al., 2003). The past few years have witnessed significant advances in drug discovery technologies for the identification of novel lead compounds against a wide range of therapeutic targets including chemical matter to disrupt protein-protein interactions, which is notoriously difficult to design (Dang et al., 2017; Lai and Crews, 2017; Neri and Lerner, 2018). Among these advances, two complementary computational approaches have been developed including (1) a protein structure-based (target-centric) approach, which utilizes the knowledge of the three-dimensional structure of the protein as well as its ligand binding pocket features (Andricopulo et al., 2008; Lionta et al., 2014; Sarkar and Goswami, 2018); and (2) a ligand-based (ligand-centric) approach, which relies exclusively on the variation of biological response with diverse chemical structures, and comprises Pharmacophore and Quantitative Structure-Activity Relationship (QSAR) approaches (Brown, 1998; Basith et al., 2018).

Small molecule drugs have been shown to bind on average to a minimum of 6-11 distinct targets excluding their intended pharmacological target (Metz and Hajduk, 2010; Peón et al., 2017). These additional targets are typically referred to as off-targets and interactions with these targets can be potentially adverse in nature (Bantscheff et al., 2009; Scheiber et al., 2009). The off-target interactions are generally weaker in affinity than those with the intended pharmacological target, but may be relevant in cases of higher cellular expression of the off-target or high systemic exposure, such as in preclinical toxicity studies (where higher doses are interrogated to define the toxic profile of the compound), clinical mis-dosings, accidental or intentional overdose, drug-drug interactions (which may lead to higher systemic exposures), or other unanticipated individual variations (which also can lead to higher systemic exposures) (Whitebread et al., 2016). The number of off-targets for a small molecule is probably always significantly higher than what is being reported at the time of a marketing application because pharmaceutical companies profile compounds using a predefined (restricted) non-overlapping target panel consisting of a limited set of targets (typically <100) (Lynch et al., 2017). An Innovation and Quality (IQ) consortium survey conducted by the Drusafe leadership group indicated that preclinical toxicities represent more than one third of the causes for drug safety-related attrition in pharmaceutical research and development (R&D). Notably, this survey indicated that the top reason for drug attrition was non-clinical toxicity due to off-target interactions (Ralston, 2017).

Consequently, an improvement in the prediction of off-target interactions represents an opportunity to improve the probability of success through a decreased preclinical safety-related attrition rate. Therefore, an integrated computational process that predicts potential off-targets and associated outcomes for small molecules was developed. This computational framework is easily extendable to facilitate the interoperability of new and existing off-target prediction methods and is programmable to automate repetitive and systematic computations. To evaluate this integrated computational framework, the methods were tested using carefully selected discontinued and Food and Drug Administration (FDA) approved drugs to assess whether they would predict reported known interactions. In addition, 15 internal molecules with well-characterized off-target interactions were evaluated.

## Methods

### OTSA Framework

**Figure 1:**
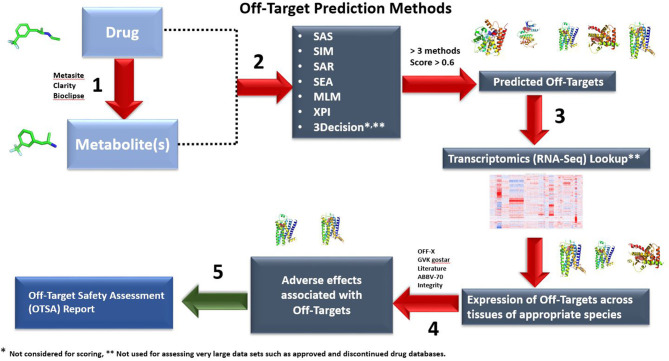
Outline of the OTSA computational screening of small molecule drugs using multiple computational models. By assessing drugs and metabolites with multiple tools simultaneously, safety scientists can screen *in silico* beyond current off-target binding and kinase screening panels. Both 2-D (six methods) and 3-D (3Decicion) methods are used to produce a list of potential off-target interactions. The predicted targets with computed scores >0.6 and above in at least 3 out of the 6 prediction methods will be moved to Step 3. These are then compared with the “body atlas” GTEX (human) and internal RNA-Seq data from untreated species (rat, monkey and mouse) to predict potential target tissues (Step 3). In addition, outcome prediction tools are used to predict the consequences of predicted interactions (Step 4). In step 5, an OTSA comprehensive off-target pharmacology report is generated. Off-target interactions considered to be of potential consequence are verified and evaluated using appropriate *in vitro* or *in vivo* models. In the current manuscript, for the approved and discontinued drugs as well as our 15 internal compounds, only steps 1 and 2 were used because of throughput limitations. Steps 3 to 5 are used to contextualize the off-target prediction and predict potential target tissues as well as biological outcomes.

The overall OTSA computational process is described in Figure 1. This computational framework uses hierarchical computational methods including simple two-dimensional (2-D) chemical similarity, Similarity Ensemble Approach (SEA), local QSAR (Quantitative Structure Activity Relationship), three-dimensional (3-D) surface pocket similarity search, automated molecular docking and machine learning algorithms such as artificial Neural Network (aNN), Support Vector Machine (SVM), and Random Forest (RF). In practice, the OTSA process predicts *in vitro* and *in vivo* testable off-target binding targets that may be adverse as well as therapeutic.

### OTSA Workflow

The full OTSA workflow is comprised of a number of 2-D target prediction methods and 3-D protein structure-based approaches including 3Decision, a web-based protein structure application utilizing public as well as proprietary structures, co-developed by AbbVie, Inc. (North Chicago, IL) and Discngine S.A.S (Paris, France). The predicted off-targets from these orthogonal target prediction methods are ranked using normalized pseudo-score, which is the sum of the linear combination of scores from the different ligand-centric methods. Pseudo-score of 1.00 reflects certainty: it is assigned when the test molecule is present in the training set with that mechanism of action (MoA) or if most of the methods predict a particular target as a likely binding partner. In order to choose reasonable pseudo-score cut-off values, 15 well-studied marketed kinase inhibitors were used as a training test. The predicted score values for these inhibitors were analyzed within the context of available pharmacological as well as other off-target information. Most of the known off-targets, as well as the pharmacological target were predicted with a score value ≥0.6 (Table 1). Thus, pseudo-score of ≥0.6 was set as significant for the present off-target analysis for approved, discontinued and internal compounds. The score from the 3Decision approach is not considered in the score normalization, because 3Decision assesses each putative protein-ligand complex using a combination of geometric and energy terms that includes geometrical features such as shape, size, binding site length, width, depth, hydrophobic patches, nature of amino acid residues, number of hydrogen bond donor and acceptors and interaction energy. In a typical OTSA process, the 3Decision results help to further confirm the results from the 2-D methodologies.

**Table 1:** Selected kinase inhibitors used to establish the pseudo-score threshold.

**Drug**	**Primary target(s)**	**Predicted score(s)**
Dasatinib	Bcr-Abl, KIT, Src, Ephrin receptor	0.86, 0.76, 0.6, 0.81
Ponatinib	Bcr-Abl, Src	0.64, 0.71
Bosutinib	Bcr-Abl, Src	0.64, 0.89
Pazopanib	KIT, FGFR, VEGFR1, PDGFR	0.78, 0.71, 0.85, 0.78
Lapatinib	EGFR	0.86
Regorafenib	Tie	0.86
Ibrutinib	BTK	0.89
Gefitinib	EGFR	0.86
Ruxolitinib	JAK1, JAK2	0.85, 0.85
Nilotinib	Bcr-Abl	0.71
Sorafenib	VEGF, PDGFR, B-Raf	0.81, 0.6, 0.71
Vemurafenib	B-Raf	0.6
Sunitinib	VEGFR, PDGFR, and other Tyr kinases	0.6, 0.78
Imatinib	Tyrosine kinases –multiple; Abl, KIT	0.71, 0.6
Crizotinib	MET, ALK	0.89, 0.85

The key steps involved in the off-target prediction workflow are described below:

Step 1: Potential Phase I and Phase II metabolites are predicted for the small molecule of interest. If *in vitro* metabolite profiling is available, these data are combined with the predicted metabolites to create a Meta-List. Metasite (Molecular Discovery, Hertfordshire, United Kingdom), Bioclipse (Spjuth et al., 2007), and the metabolite prediction module of the Clarity suite (Chemotargets, Barcelona, Spain) were used for prediction of phase I and II metabolites. The bioclipse tool can identify possible sites of metabolism in the preclinical species including rat and dog.

Step 2: 2-D chemical similarity and machine learning methods (MLMs) were used to profile potential targets for both the parent molecule and its metabolites. The six different cheminformatic methods used in the present study are Similarity Active Subgraphs (SAS) (Willett, 2009), SAS based QSAR models (SAR) (Gregori-Puigjané and Mestres, 2008), Molecular Similarity (SIM) (Metz and Hajduk, 2010), SEA (Keiser et al., 2009), and Cross Pharmacology Indices (XPI). The MLMs include RF, aNN, and SVM (Kotsiantis et al., 2007). Below, each six cheminformatic and 3-D methodology used in the OTSA process are described.

- SAS: Given a biological target, a SAS is defined as the simplest active subgraph containing the minimum pharmacophoric features needed (i.e., structural features) to achieve activity. SAS screening allows for the identification of similar pairs of molecules previously categorized as dissimilar, expanding the applicability domain and improving prediction (Willett, 2009). In addition, the SAS methodology prevents the identification of the vast majority of similarity artifacts and reduces the impact of false positives, thereby improving the precision.
- SAR: SAR facilitates the development of large-scale QSAR models (Gregori-Puigjané and Mestres, 2008) for each target family (e.g., kinases, GPCRs, ion channels, proteases, transporters, immunoglobulin receptors, and others).
- SIM: Three types of 2-D descriptors are used: (1) Pharmacophoric Fragments (PHRAG), (2) Feature-Pair Distributions (FPD), and (3) Shannon Entropy Descriptors (SHED). PHRAG and FPD calculate the similarity between two chemical structures that account for the overlapping portion of their active profile (Mestres et al., 2006; Vidal et al., 2011). SHED computes the similarity between two molecules using Euclidian distance (Vidal and Mestres, 2010). Thus, each descriptor characterizes chemical structures with a different degree of randomness that complements each other in terms of chemical structural similarity.
- SEA: The similarity ensemble approach was originally developed by Keiser et al. (2009) to identify related proteins based on the setwise chemical similarity among the ligands. SEA has proven its ability to predict novel ligand-target interactions using chemical structure information alone (Lounkine et al., 2012).
- MLM: More than a thousand high-quality aNN, SVM, and RF classifiers were generated based on FPD molecular descriptors for qualitative binding prediction (Clarity Chemotargets, Barcelona, Spain). MLM is a consensus score of the three MLMs; if the consensus score results are positive, the corresponding ligand-target link is considered to be likely.
- XPI: This public domain availability of cross-pharmacological data for thousands of small molecules on many different biological targets enables an in-depth cross-pharmacology analysis (i.e., link small molecules to potential off-targets) (Schmidt et al., 2014).
- The target profiling module in 3Decision utilizes a combination of three different methods. These are 3-D methods or receptor-based (e.g., pocket sequence, pocket properties), 2-D methods or ligand-based (e.g., ligand fingerprint, ChEMBL profiling) and lastly a protein-ligand interaction pattern. For the current work, we focused on using only 2-D methods. The first method is the ligand fingerprint based, in which Extended-Connectivity Fingerprints (ECFP6) was used to calculate tanimoto similarity between the probe molecule and the ligands extracted from all public and proprietary X-ray crystal structures. The second method is the ligand pharmacophore which utilizes a fuzzy 3D pharmacophore representation of ligands to compare a pair of ligands. This method does take the conformation of the ligands in account and calculates root mean square deviation (RMSD) of superimposition. The third and last method in the 2-D category is ChEMBL profiling, which calculates the similarity between compounds from various assays and the probe molecule. Users can choose the sensitivity of these searches and ensure stringent or loose searches.
  The top scoring off-targets from this 3Decision (3-D binding site comparison search approach) step are generally combined with the 2-D predicted off-targets (six methods) to create a master off-target list (component 2 of OTSA process). This step was not used for the approved and discontinued drug lists evaluated in the current study, as the 3Decison method is not high-throughput and would not be practical for such a large collection of molecules. However, for the internal 15 compounds, the 3-D method (3Decsision) was used in addition to the 2-D methods.

### Physicochemical Properties

Several studies indicated the importance of physicochemical properties of small molecules in drug attrition and molecular promiscuity. There have been many rules reported to minimize toxic events, chemical promiscuity or optimize oral absorption, including: (1) compounds satisfy both clogP [octanol to water partition coefficient) ≤3 and topological polar surface area (TPSA) ≥ 75A°2 are said to be in full compliance with the 3/75 rule, Hughes et al. (2008); (2) compounds with less basicity (pKa < 5) (Luker et al., 2011); (3) Lipinski's rule of 5/Ro5 (i.e., molecular weight (MW) < 500, logP < 5, hydrogen bond donors (HBD) < 5 and total number of hydrogen bond acceptors (HBA) < 10] (Lipinski et al., 1997); (4) increased number of chiral carbon atoms (sp^3^count) to increase molecular complexity (Lovering et al., 2009); and (5) less rotatable bonds to decrease entropic contribution (Veber et al., 2002). Generally, oral small molecule compounds complying with these rules have a higher probability of success. For approved, discontinued and internal compounds, the Qikprop (Schrodinger, New York, NY) and AIDEAS (Gupta, 2015) tools were used to compute many of these physicochemical properties.

### Testing Sets of Compounds

**Figure 2:**
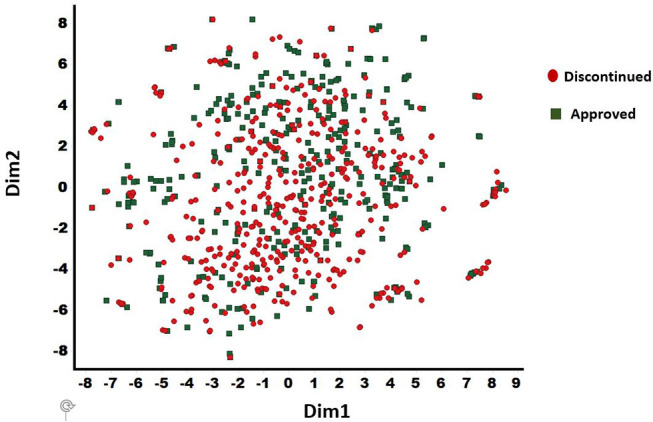
t-SNE method derived chemical diversity distributions for 857 drugs (401 approved and 456 discontinued): 25 clusters are shown. Only the relative distance between molecules in 2-D projection (each spheres) is meaningful. X (Dim1) and Y (Dim2) are the projections in 2-dimensions from a multidimensional descriptor space. The discontinued and approved drugs are shown in filled circle and diamond shapes, respectively.

The discontinued and FDA approved drugs were obtained from the Cortellis (Clarivate, Philadelphia, PA), Citeline (San Diego, CA), and FDA (Silver spring, MD) databases. Overall, 457 discontinued and 401 approved drugs were selected based on the consistency of curated data associated with each compound in these databases. The BindDB database (Gilson et al., 2015) has a collection of FDA approved drug candidates together with associated *in vitro* data. Thus, this dataset was used for our computational study. For discontinued drugs, we used two data sources (Cortellis and Citeline—as of June 2018). A compound was selected for the current work as discontinued only if both the Cortellis and Citeline databases were in agreement. The diversity analysis was performed for the combined dataset of 857 compounds (401 approved and 456 discontinued) using a t-distributed stochastic neighbor embedding (t-SNE) machine learning approach (van der Maaten and Hinton, 2008) to confirm the presence of diverse chemistry. This provided 25 distinct clusters with a clean separation among various chemotypes within the clusters (Figure 2). The t-SNE machine learning method generates a mapping from a multi-dimensional space (i.e., physicochemical property space) to a 2-D space (dim1 and dim2) and in this visualization only the relative distance between molecules in 2-D projection (each spheres) is meaningful. Additionally, 15 internal small molecules were selected from AbbVie's compound collection. These 15 compounds were selected based on the availability of sufficient off-target data generated through traditional lead optimization screens (i.e., secondary pharmacological counter-screens; CEREP radioligand displacement assays) as well as diversity in terms of chemistry and pharmacology. These compounds were screened against a panel of 70 targets with radioligand displacement assays (AbbVie 70 panel; Eurofins, Celle L'Evescault, France) (Lynch et al., 2017). A target in this panel is considered as a potential hit only if the displacement is >50% at a 10 μM compound concentration.

The 3-D structures of these compounds were generated using the ligprep module within the Maestro tool (Schrödinger, New York, NY). The processed molecules were then used to compute a wide range of physicochemical properties using AIDEAS (Gupta, 2015) and Qikprop (Schrödinger, New York, NY) and visualized by Spotfire (TIBCO Inc., Palo Alto, CA). MDCK and Caco-2 cell permeability values were computed using Qikprop. All other physicochemical properties were computed using the AIDEAS tool. Specifically, MW and selected physicochemical properties (clogD, clogP, AlogP), TPSA, pKa, number of rotatable bonds, number of sp^3^ carbon atoms, hydrogen bond acceptors and donors) were evaluated, since these have been shown to be associated with a higher degree of promiscuity (Blomme and Will, 2015). Finally, these molecules were processed through the OTSA workflow to generate a list of likely off-targets in order to compare with the off-targets identified *in vitro* for these compounds.

### Statistics

One-way analysis of variance (ANOVA) was performed for each of the computed physicochemical properties of the approved and discontinued drugs as well as the 15 internal discovery stage compounds. Additionally, Tukey Honest Significant Difference (HSD) *post-hoc* tests (Six et al., 2000) were applied to present multiple pairwise-comparisons. For each comparison, a pairwise Welch's correction (Welch, 1951) was added, as this test does not assume homogeneity of variances within the descriptors. Pairwise comparisons using the non-parametric Wilcoxon rank sum test (Wilcoxon et al., 1970) were also computed, as this distribution free test does not assume the data (i.e., descriptors) to follow a specific distribution pattern in contrast to a standard ANOVA. Significance and differences in the datasets were corrected for multiple testing based on the Bonferroni-Hochberg procedure (Benjamini and Hochberg, 1995) at a False Discovery Rate (FDR) of 5%. Data distributions from these tests were visualized via violin plots (Hintze and Nelson, 1998).

## Results

### Physicochemical Properties of the Approved, Discontinued, and Internal Molecules

The computed mean and median of the physicochemical properties for the selected 456 discontinued, 401 FDA approved, and 15 internal test compounds are compared in Table 2. ANOVA statistical comparisons on each of these properties for the approved and discontinued drug categories are also summarized in Table 2, while the distributions of each physicochemical property are visualized by violin plots (Supplementary Data Sheets S3A–J). The computed clogD (distribution coefficient), clogP, MW, logS (aqueous solubility), number of rotatable bonds, pKa and number of hydrogen bond acceptors were statistically different between the approved and discontinued drugs in all the statistical tests including one-way ANOVA and Welsh statistics (Supplementary Data Sheet S1). ANOVA assumes equal variances within the descriptor space. However, the computed Madin-Darby Canine Kidney (MDCK) cell permeability, Caco2 cell permeability, topological polar surface area (TPSA), acidic features, number of sp^3^ carbon atoms, sum of hydrogen bond donor and acceptors were not statistically different between the approved and discontinued drugs. The computed sp^3^ carbon atom count, MW, HBD, HBA, number of rotatable bonds, TPSA and pKa (basic as well as acidic) of the 15 internal compounds were also not statistically different from those of the FDA approved drugs.

**Table 2:** Comparison of computed physicochemical properties for approved, discontinued, and discovery drugs.

**Computed properties**	Approved drugs (mean, median, SD^*^)	**Discontinued drugs (mean and median, *SD*)**	**AbbVie compounds (mean, median, and *SD*)**	***p*-value from ANOVA for approved vs. discontinued drugs**
Molecular Weight	339.3, 315.6, 120	379.8, 375.6, 104	352.6, 357.7, 67	0.0000005
logP	1.88, 2.38, 3.1	2.9, 3.2,2.7	3.9, 4.1, 1.5	0.00001
TPSA	71.3, 63.3, 44	73.9, 68.9, 42	62.2,60.7,17	0.64
HBA	4.3, 4.0, 2.8	4.5, 4, 2.7	4.2,5,1.4	0.05
HBD	1.72, 1,1.8	1.68, 1,1.8	1.4, 1, 1.2	0.88
No. rotatable bonds	5.1, 4.0, 4.0	5.49, 5.0, 3.0	4.2, 4, 1.3	0.11
sp^3^ count	7.99, 7.0, 6.1	7.66, 7.0, 4.9	5.8, 6.0, 1.8	0.62
Caco2 nm/s	980.10, 368.09, 1,325	773.7, 359.7, 1,108	1928.8, 1037.9, 1,648	0.98
MDCK nm/s	960.10, 312.90, 1,657	999.8, 327.7, 1,849	2686.9, 1332.2, 2,824	0.47
logS	−2.08, −2.0, 2.2	−4.54, −4.5, 2.0	−5.2, −4.8, 1.7	0.000000001

SD

* *, standard deviation. The computed p-values from ANOVA between approved and discontinued drugs is listed in the last column*.

**Figure 3:**
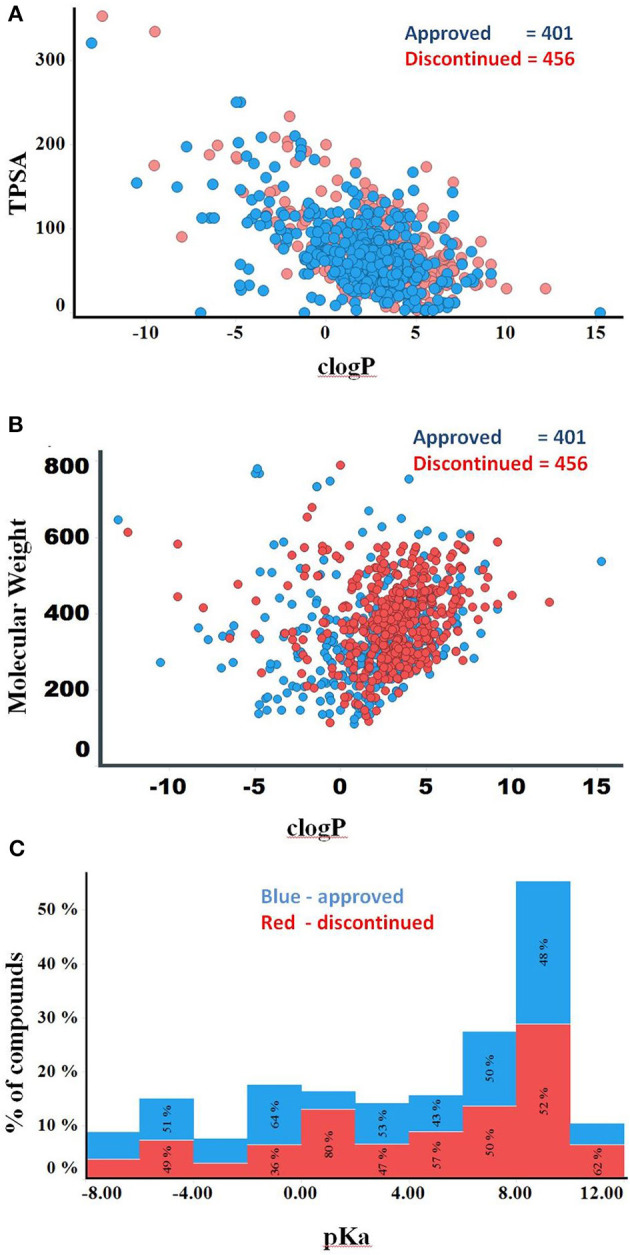
**(A)** Plot showing the clogP vs. TPSA distribution for approved (blue) and discontinued drugs (red). **(B)** Plot showing the clogP vs. MW distribution for approved (blue) and discontinued drugs (red). **(C)** Distribution of pKa values of the discontinued and approved drugs. Computed pKa values are shown on the X-axis. The Y-axis shows the number of compounds in each pKa bin. Red and blue color bars indicate discontinued and approved drugs, respectively.

Compounds with clogP ≤ 3 and TPSA ≥ 75A°2 are said to be in full compliance with the 3/75 rule. A total of 120 (26%) and 121 (30%) of the discontinued and approved drugs satisfied the 3/75 rule. To obtain a complete perspective on these properties, compounds were further binned into the following 4 categories: (1) TPSA ≥ 75 and clogP ≤3; (2) TPSA <75 and clogP > 3; (3) TPSA ≥75 and clogP > 3; and (4) TPSA <75 or clogP ≤3 (Table 3). Figure 3A compares the TPSA and clogP distributions for approved and discontinued drugs, reflecting somewhat similar distributions for these drug categories. Figure 3B compares the clogP and MW space for approved and discontinued drugs, which also indicates a high degree of similarity. In particular, in this space, 319 (76%) and 313 (70%) of the discontinued and approved drugs were in full compliance with the Ro5 (i.e., no violation), indicating similar distributions for both drug categories.

**Table 3:** Approved and discontinued drugs within the 3/75 physicochemical space.

**3/75 Criteria**	**Approved (%)**	**Discontinued (%)**
TPSA≥75; clogP ≤3	30	26
TPSA <75;cLogP>3	28	37
TPSA≥75; cLogP>3	9.2	17
TPSA <75;cLogP ≤3	31	37
TPSA≥75	39	43
cLogP ≤3	62	44

*Both clogP and TPSA criteria are met for only 30 and 26% of approved and discontinued drugs, respectively*.

The higher the pKa value, the stronger the basicity associated with a compound. Basic pKa values, primary, secondary and tertiary amines generally carry a positive charge depending on the atomic environment where they are located. Figure 3C shows the computed pKa (reflective of basicity) value distribution for the approved and discontinued compounds, indicating a slightly greater number of compounds with a pKa value between 7 and 10 in both categories. In total, 29% (120) of approved and 32 % (147) of discontinued drugs have a pKa value between 7 and 10 at a physiological neutral pH value. The computed adjusted *p*-value using ANOVA, statistical test for pKa between the approved and discontinued drugs were 0.03, indicating that the pKa values are statistically different between these drug categories (Supplementary Data Sheet S1).

Taken together, these results overall show statistically significant differences in certain computed physicochemical properties such as basicity (pKa), MW, clogD (or clogP), and logS between the approved and discontinued drugs. The computed AlogP, clogD, sp^3^ count, and logS values were also statistically different between the FDA approved and the selected 15 internal compounds.

### Predicted Off-target Interactions for the Approved and Discontinued Drugs

The structural neighbors are the ones that share the structural and pharmacophoric features with the query chemical structure. Chemical similarity methods alone identified 28152 and 47868 compounds as structural neighbors (or chemically similar) to the 401 approved and 456 discontinued drugs, respectively. However, the inclusion of pharmacophoric and fingerprint features (PHRAG, SHED and FPD fingerprint descriptors) identified 55510 and 159210 compounds as nearest neighbors from the database, indicating that these additional features captured a greater number of similar compounds from the integrated database for the target prediction process. The SIM, SEA and XPI target prediction methods use 28152 and 47848 structural neighbors for prediction of targets for the approved and discontinued drugs, respectively. The other 3 methods (SAS, SAR, and MLM) use a larger number of chemically similar compounds (55510 and 159210) for the approved and discontinued drugs.

**Figure 4:**
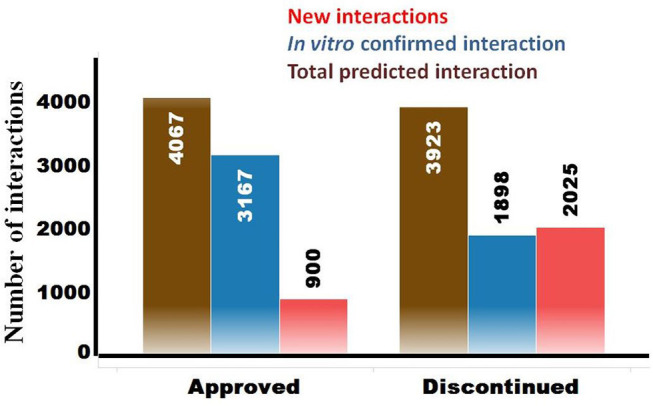
Summary of predicted high-scoring interactions for the approved (*n* = 401) and discontinued (*n* = 457) drugs. The total predicted off-targets for each category are shown in brown. The *in vitro* confirmed interactions are in blue, while the novel yet to be confirmed interactions are in red.

The OTSA results for the 857 compounds (401 approved and 456 discontinued drugs) are summarized in Table 4. This computational process resulted in a total of 7,990 high-scoring interactions for all compounds, translating to 9.3 interactions per compound. The predicted off-target interactions for the approved and discontinued drugs indicated an average of 10.1 and 8.6 interactions per compound, respectively, suggesting a higher promiscuity for the former compared to the latter. Comparison of these predicted high-scoring interactions with existing *in vitro* data showed that 22% (900) and 51.5% (2,025) were new (i.e., not previously reported or not previously confirmed) interactions for the approved and discontinued drugs, respectively (Figure 4). In other words, 78 and 49% of the predicted interactions for the approved and discontinued, respectively, have been previously confirmed *in vitro*. Overall, the OTSA process identified an average of 2 and 4 high-scoring new interactions for the approved and discontinued drugs, respectively. It should be noted that more than 90% of these new interactions were predicted by at least by 4 orthogonal target prediction approaches used in the OTSA process, suggesting a high confidence of the prediction.

**Table 4:** Summary of the predicted interactions for the approved drugs, discontinued drugs, and AbbVie compounds.

**Drug status**	**No. Drugs**	**Total predicted interactions**	***In vitro* confirmed interaction**	**New interactions (yet to be confirmed)**	**Ave. interactions/drug**
Approved	401	4,067	3,167	900	10.1
Discontinued	456	3,923	1,898	2,025	8.6
AbbVie	15	123	70	53	8.2

Further analysis on the predicted interactions of the approved and discontinued drugs revealed that 27% (1,107 interactions) and 12.8% (504 interactions), respectively, were part of the training set, indicating that more than 73% of the interactions (i.e., not present in the training set) were predicted using ≥3 target prediction methods (Supplementary Data Sheets S2A,B). A pseudo score of 1 is assigned to the training set interactions. Additionally, these training set interactions with a score of 1 were not considered while evaluating the performance of the OTSA predictions (Supplementary Data Sheets S2A,B).

Of the total 4,067 interactions for the approved drugs, 2,960 interactions were predicted, and the remaining 1,107 interactions were part of the training set. Among these 2,960 predicted interactions (i.e., 4,067–1,107), a total of 2,502 (84%), 2,319 (78%), 2,433 (82%), 2,124 (72%), 2,391 (81%), and 2,211 (74%) interactions were predicted by SAS, SAR, SIM, SEA, MLM, and XPI methods, respectively. Interestingly, 923 of 2,960 (i.e., 31%) interactions were predicted by all the six methods. Of these, 718 and 205 are confirmed and new interactions, respectively.

Among the total of 3,923 interactions for discontinued drugs, 504 interactions are part of the training set. An additional analysis on the other remaining 3,419 interactions revealed that a total of 2,772 (81%), 2,653 (77%), 2,522 (73%), 2,369 (69%), 2,304 (67%), and 2,437 (71%) interactions were predicted by SAS, SAR, SIM, SEA, MLM, and XPI methods. All the six methods predicted a total of 706 of 3,419 (i.e., 24%) interactions. Of these, 479 and 227 are confirmed and new interactions, respectively.

Supplementary Table S1 summarizes the number of predicted interactions for the approved and discontinued drugs from the different methods. For both the approved and discontinued drugs, the SAS method performed slightly better compared to the other methods in identifying greater number of interactions (2502 of 2940 for approved and 2772 of 3419 for discontinued). The computed pseudo score range for these predictions is 0.65–0.91 for the approved drugs and 0.68–0.94 for the discontinued drugs.

The analysis also showed that 600 predicted off-targets were common to both the approved and discontinued drugs (Figure 4), supporting that interaction with these off-targets may not have played a major role in the termination of the latter. Of these 600 common targets, 508 (84%) and 351 (58.5%) are confirmed *in vitro* off-targets for the approved and discontinued drugs, respectively.

**Figure 5:**
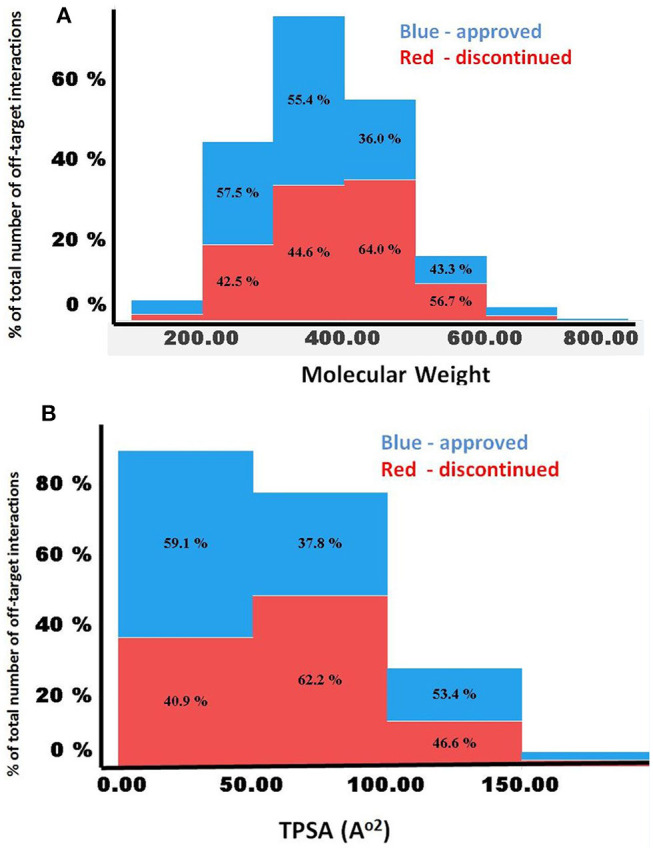
**(A,B)** Comparison of MW and TPSA with number of predicted off-targets for the approved and discontinued drugs. The X-axis shows **(A)** MW and **(B)** TPSA. In both figures, the Y-axis shows the percentage of predicted off-targets in each of the bins.

Figures 5A,B compares the predicted off-targets for the approved and discontinued drugs within the context of MW, clogP, and TPSA, since these physicochemical descriptors have been shown to be associated with a higher degree of promiscuity. Interestingly, for both drug categories, higher promiscuity was observed for compounds with a MW range of 300–500, TPSA of ~200, and clogP ≥7. This distribution also indicated significantly lower promiscuity (i.e., number of off-targets) for compounds with MW>700 and MW <200 for both categories, indicating a range for these properties that are linked to specificity of the molecules. Nevertheless, the computed physicochemical properties did not provide any reasonable estimate of the predicted number of off-targets or promiscuity.

### Unique High-Scoring Off-Targets for the Approved and Discontinued Drugs

Further data interrogation identified 325 and 286 high-scoring predicted targets unique to the approved and discontinued drugs, respectively. Importantly, ~88% (285 out of 325 targets) and ~ 51% (146 out of 286 targets) of these interactions for approved and discontinued drugs were confirmed *in vitro*. Comparison of these key predicted off-targets of approved and discontinued drugs indicated that certain cytochrome p450 (CYP) isoforms(1A2,1A1,2D6,2A6,2C9), T, N, and L type calcium channel voltage-dependent channels, sodium channels subunit α isoforms (1–9), cyclin-dependent kinase (CDK) isoforms (2, 5, 6, 8, 9), Phosphodiesterase 3 (PDE3), and hERG K^+^ channel are common off-targets for the discontinued drugs, but not for the approved drugs. Taken together, these results suggest that interactions with these targets decrease the overall probability of success for compounds.

### Internal Test Compounds

To further test the strengths and limitations of the OTSA process, 15 well-studied internal compounds were evaluated and data compared with available *in vitro* CEREP or other off-target data. The primary and secondary *in vitro* confirmed targets for these selected 15 compounds are summarized in Table 5. The OTSA process predicted several of these targets identified *in vitro* with a high score for each compound. Encouragingly, the correct pharmacological target was predicted as one of the top scoring hits (i.e., within the top 3 ranked) for 14 compounds. For A-967079, the pharmacological target (i.e., Transient receptor potential cation channel, subfamily A member 1 or TRPA1) was not predicted as a top target. However, this target was predicted with pseudo-score of 0.5, which is slightly below the set threshold score of 0.6. Finally, the 3Decision based search approach independently identified the primary pharmacological targets for all 15 compounds.

**Table 5:** Comparison of the predicted off-targets with the *in vitro* binding data for the 15 internal molecules.

**Compound ID**	***In vitro* off-targets**	**Predicted off-targets**	**Pharmacological target**
A-1390577	*Cav1.2, CDK5,8*	*Cav1.2*, KCNH2, 5HT_2b_, BRAF, *CDK5,8*	PKC Predicted score 0.8
A-1411735	*Cav1.2*, Opioid κ	*Cav1.2*, KCNH2, HTR_2A_	PKC Predicted score 0.81
A-1593308	D2, Nav1.5, Cl^−^ and *Cav1.2*, A2A, PPARγ, COX2, *SMO*	*SMO, Cav1.2*,HSDB11, ITGAL, *D4*, CYP3A4	SGPL1 Predicted score 0.6
A-317567	*5HT(2b,2a,1a,5a), opioid*, σ, μ, δ, M2, M3, M4, DRD, α1 and 2, BZD	*5HT(2a,2b,1b,2b,1d 5a,b), opioid* μ, κ, *M2*, α*2*, CB1, BCL2, A1, Cav1.2	ASIC Predicted score 0.99
A-438079	BZD	CB2, TRPV1,opioid κ	P2RX7
A-777903	D4, *D3*, D5, *5HT(2b*,2a,1a,3,5a,*7*),*Nav1.5*, Cav1.2,*opioid* σ, opioid κ, opioid μ, β1,2, *H2*, α*2, DRD*	*M2, opioid* μ*, opioid* σ, *Nav1.5*, α*2b, 5HT (1a,2b,2c,7), H1,D3*, KCNH2, SERT	Melanin receptor Predicted score 0.76
A-803467	A3, A2A,BZD, *5HT_2_*_a_, D1, CB1, opioid μ, H2	SCN3a, SENP7, α1a, *5HT_2*a*_*, H1, D2	Nav1.8 Predicted score 0.62
A-836339	A3, 5HT_2c_	CB1,5, opioid κ, TRPV1, 5HT_2b_, Nav1.7,A1	CB2 Predicted score 0.66
A-889425	*Opioid* σ, Cl^−^ and Nav1.5, *D4, 5HT_2*c*_*	*5HT_2*c*_, D4, Na 1.7, opioid* σ	TRPV1 Predicted score 0.66
A-922500	BZD	KCNH2, CDK8	DGAT1 Predicted score 0.71
A-967079^+^	D2 (43%)	CYP2A13, TRPV1, 3, 4	TRPA1 Predicted Score 0.5
A-970781	*A2A* (23%)	mGlu5, *A1*	DGAT1 Predicted score 0.98
A-993610	opioid κ	Opioid μ, CB1, H3	TRPV1 Predicted score 0.74
A-995662	*PPAR*γ *(38%)*	KIT, CYP3A4, CYP2D6, PPARγ, KDR, P2RX7, Cav1.2	TRPV1 Predicted score 0.78
A-908292	–	KCNH2, ROCK1, EP4, 5HT_2c_, CB1, PPARα	ACC2 Predicted score 0.77

^+^*The pharmacological target TRPV1 was not predicted with score threshold of 0.6*.

*The second and third columns show the targets identified by CEREP and OTSA, respectively. The fourth column shows the pharmacological target along with the pseudo-score*.

Altogether, the OTSA predicted 123 interactions for these 15 compounds, indicating an average of 8.2 interactions per compound, a value slightly lower than the polypharmacology distribution predicted for the FDA approved drugs as described above. Comparison of the *in vitro* CEREP binding data with the *in silico* predicted targets indicated that ~56% of the predictions overlapped, which implies that 44% of known interactions were not predicted.

## Discussion

Computational prediction of toxicity has been a desirable objective of toxicologists for decades, but it is only in the last few years that significant advances have been made through the integration of information and data from multiple scientific disciplines, including chemistry, biology, pharmacology, genomics, and basic toxicology (Bai and Abernethy, 2013; Luechtefeld et al., 2018). This integrated framework offers the opportunity to transform data into useful knowledge for the toxicologist. One approach to predict toxic effects is to generate a better understanding of off-target interactions together with a comprehensive analysis of physicochemical properties for small molecules. These predictive computer approaches generally take advantage of key strengths from each of the above mentioned diverse disciplines. Several computational methods have been developed to predict off-targets and associated toxicities for small molecules, such as: (1) Swiss Target Prediction (Gfeller et al., 2014), (2) Chemical Similarity Network Analysis Pull-down (CSNAP) (Senese et al., 2014), (3) SuperPred (Dunkel et al., 2008), (4) Search Tool for Interacting Chemicals (STITCH) (Kuhn et al., 2013), (5) SEA (Keiser et al., 2009), (6) Tarpred (Liu et al., 2015), (7) Chemotargets (Gregori-Puigjané and Mestres, 2008), (8) Prous Institute Symmetry (Prous, 2016), (9) Pocket Similarity Search using Multiple-sketches (PoSSum) (Ito et al., 2011), and (10) PSILO (Feldman and Labute, 2010). Overall, these methods predict a number of interactions using a specific pair of method/database combinations. However, these computational methods offer different flavors of ligand 2-D similarity and protein binding site similarity searches together with focused databases, each with its unique strengths and weaknesses. Consequently, the off-target predictions from each of these methods significantly vary as they are utilizing different annotated databases and similarity search algorithms. This variability compelled us to develop a framework called OTSA that assembles several of these computer-based off-target prediction methods together.

Drug molecules induce toxicity through various mechanisms, including interactions with unintended cellular off-targets in different tissues with varied binding strengths. The identification of these off-target interactions (and relative binding strength to these off-targets) in various tissues can help determine the potential toxicological liabilities of small molecule drugs. Screening every compound against a large portion of the human proteome at the lead optimization stage is not practical from a time and resource perspective. Current *in vitro* testing paradigms at the lead optimization stage are typically limited to those targets with a close relationship to the intended target (e.g., isoforms) or panels of off-targets selected based on their promiscuity or safety relevance (Blomme and Will, 2015). For example, off-targets like hERG, 5-HT_2b_ or PPARγ, which are known to be associated with safety liabilities, are often used in lead optimization stage counter-screens. Given the current limitations, *in silico* off-target prediction approaches have been receiving more attention in the past few years, mostly to understand polypharmacology and target engagement associated with preclinical and clinical toxicities (Lavecchia and Cerchia, 2016; Van Vleet et al., 2018; Zloh and Kirton, 2018). These *in silico* methods are now actively implemented at the early discovery and preclinical drug development stages for rapid generation of off-target binding hypotheses.

### Uniqueness of the OTSA Platform

The OTSA platform rapidly generates adverse off-target interaction hypotheses by using both ligand- and target-centric methodologies. The latter method automates high throughput 3-D target-model building, binding site identification, as well as conformational analysis, docking, scoring, and ranking small molecule ligands, while the former utilizes an integrated chemistry database framework consisting of the SAR profile for over 7,000 (~35% of the human proteome) targets. This integrated approach significantly differs from other published approaches in many ways as described below.

First, this approach uniquely combines predicted and *in vitro* identified metabolites as part of the off-target prediction process. This step is an important component, as preclinical toxicities and clinical safety events may be related to metabolite interactions with unintended off-targets (Whitebread et al., 2016). For example, fenfluramine was withdrawn from the market due to severe clinical valvular heart disease related to interaction by its active metabolite, norfenfluramine, with the 5-HT_2b_ receptor (EC_50_ value of 8 nM) (Setola et al., 2005). The parent drug molecule fenfluramine displayed no binding with 5HT_2b_ at 30 μM (maximum binding displacement of 4% at 30 μM). Interestingly, our computational process not only predicted norfenfluramine as a metabolite, but also its likely interaction with 5HT_2b_. However, the OTSA process also predicted 5HT_2b_ as an off-target for the parent fenfluramine. Another example is the internal test compound, A-908292, for which the top predicted metabolite was flagged to interact with PPARα, a prediction consistent with *in vivo* rat data (Waring et al., 2008). This may also be the reason for differences in OTSA-predicted and CEREP-confirmed targets.

Second, this computational workflow exploits a wide range of 2-D computational methods along with a 3-D method. Depending on the number of compounds to be profiled, a 2-D similarity search alone can be conducted or a combination, including machine learning, 3-D binding site search, and 2-D similarity search, can be used. In the present study, only the chemical similarity and machine learning approaches were used to mine the SAR datasets to predict the off-targets for the FDA approved and discontinued drug lists. 2-D methods alone have been shown to reasonably predict novel interactions. For example, Lounkine et al. (2012) have used a computational strategy (SEA) to predict the activity of some marketed drugs on 73 unintended “side-effect” targets. Close to 50% of the predicted interactions were confirmed either using databases that are unknown to the SEA method or by performing new biological assays. The measured binding affinities for these newly predicted off-targets ranged from 1 nM to 30 μM. The OTSA process effectively uses SEA as one of the 2-D methods for off-target prediction to identify such novel interactions.

Third, the OTSA computational process can be coupled with normal tissue transcriptomic profiles as part of the validation of predicted off-targets. To achieve this, the RNA-Seq-based gene expression profiles of various normal tissues of human, rat, mouse and cynomolgus monkey is coupled with the OTSA workflow. If testicular toxicity is observed in rat, for example, only the predicted off-targets that are expressed in the rat normal testis are retained, thereby refining the prediction through elimination of off-targets with little or no relevance from further *in vitro/in vivo* analysis. This feature enables the formulation of more robust hypotheses related to mechanisms of toxicity.

Finally, the OTSA process uses a large curated database that connects the predicted off-targets to preclinical toxicity and clinical safety events. At present, this database covers ~13,000 targets, translating to ~65% of the expressed human proteome. This large collection of data provides another layer of knowledge-based prediction capability.

The OTSA process has 5 components representing 5 scientific methodologies (Figure 1). Each of these methods has their own strengths. For high-throughput analyses, such as the validation work presented in this manuscript, only components 1 and 2 were used to generate target binding hypothesis for throughput reasons. However, all components (1-5) of the OTSA process are routinely used for internal late discovery and clinically advanced compounds. For example, an understanding of the expression level for the top scoring predicted off-targets across a large panel of tissues/organs of human and pre-clinical species (mouse, rat, cynomolgus monkey) is useful to better contextualize the safety risks, but also formulate hypotheses of the off-target implicated in the pathogenesis of a finding in a toxicology study. Using norfenfluramine, a key metabolite of fenfluramine, as an example, components 1 and 2 of OTSA identified eight off-targets: CYP2A6, CYP2E1, Adrenoceptor β1(ADR_b1_), Adrenoceptor α_2C_(ADRA_2c_), Adrenoceptor α_2a_(ADRA_2a_), and 5HT _(1a, 2band1d)_. Among these off-targets, CYP2E1, CYP2A6, and ADR_b1_ are part of the training set and the remaining 5 targets were predicted as well as confirmed *in vitro*. Of these, only 5HT_2b_ and ADR_2c_ are highly expressed in human blood vessels (https://gtexportal.org/home/), where this compound induces its toxicity, which eliminates 6 off-targets for further consideration in follow-up investigations. Likewise, a major challenge in the interpretation of off-target interaction data is linking target modulations to pre-clinical or clinical toxic effects. Thus, we routinely evaluate the link of off-targets to possible clinical and pre-clinical outcomes by computationally searching a set of safety databases that relate target modulation to pre-clinical/clinical outcomes (OFF-X, Bioinfogate, Barcelona, Spain). As of now, this database tracks safety liabilities associated with ~13,000 targets (>65% of proteins expressed by the human genome) and 239,766 safety alerts linked to many of these targets.

Finally, one needs to further evaluate if those safety relevant off-targets need a follow-up. This assessment relies on three criteria: (1) a strong clinical and pre-clinical evidence exists that connects an off-target interaction to a pre-clinical/clinical outcome (i.e., is the off-target interaction of toxicological relevance?); (2) tissue expression of the off-target (i.e., is the expression of the off-target in tissues of relevance?); and (3) the off-target prediction has computed scores >0.6 and above in at least 3 out of the 6 prediction methods. Normally, if the off-target satisfies these three criteria, the last component entails an in *vitro* validation or *in vivo* data interpretation.

The OTSA process uses several computational approaches e.g., OFF-X, Clarity, Transcriptomics body atlas and 3Decision. The main objective of this computational workstream is to highlight the role of different computational methodologies in predicting comprehensive off-target lists together with their normal expression in different tissues as well as literature evidence connecting off-targets to various possible outcomes. The present work validates this multi-method integration hypothesis and provides strong evidence that integrating these methodologies into a single framework provides additional utility in terms of profiling relevant off-targets for small molecules. However, the limitation of this work is that the individual components (i.e., 1-5) are not programmatically combined into one pipelined framework. This is on our roadmap to build a single framework, with a single-entry point (i.e., input chemical structure), that automatically processes all components sequentially or in user defined steps (like perform only steps 2 and 4 or 2 and 3 or 1, 2 and 3 and so on).

The purpose of the OTSA computational process is to present the consensus cheminformatics approach to predict primary and secondary off-targets for small molecules. The orthogonal cheminformatics methods used here essentially exploit different features (or fingerprints) present in the query molecule, while predicting potential off-target interactions.

Briefly, SIM, SEA, and XPI are chemical similarity-based approaches (Gregori-Puigjané and Mestres, 2008; Spitzmüller and Mestres, 2013). SEA uses a chemical similarity ensemble approach to identify related proteins based on the set-wise chemical similarity among the ligands (Keiser et al., 2009), while the SIM method uses 2-D descriptions such as pharmacophoric fragments, feature-pair distribution and Shannon entropy (Gregori-Puigjané and Mestres, 2006). XPI uses chemical similarity to mine publicly available cross-pharmacology data. The other three methods use either standard quantitative structure activity models (SAR method), simple abstraction of minimum pharmacophoric features needed to achieve some level of target activity (SAS method) or a Shannon entropy pharmacophore fingerprint information (MLM).

Overall, all cheminformatics methods performed reasonably well by capturing >65% of interactions (from a total of 2,960 and 2,772 predicted interactions for the approved and discontinued drugs, respectively). For example, for approved drugs, SAS captured 2,502 interactions out of the total of 2,960 predicted interactions (~84%). However, SEA captured only 2,124 interactions out of 2,960 interactions (~72%) for the present dataset. Similarly, for discontinued drugs, SAS captured 2,772 interactions out of the total of 3,923 predicted interactions, translating to 81% of the interactions, while SEA identified 2,304 (67%) interactions.

Beyond the consensus targets identified, those targets predicted in fewer than 3 methods may also be of relevance. However, in our OTSA process, these targets are given less confidence than those consistently predicted across 3 or more methods.

In a greater detail, the *in vitro* generated data for five internal compounds are compared with the OTSA results below.

1. **A-317567**
  A-317567 is a non-amiloride blocker of acid sensing ion channels (ASICs) (Dube et al., 2005). The OTSA process correctly identified both ASIC 1 and 3 as possible targets for this compound. Moreover, a total of 20 additional off-targets were predicted, including: (1) urokinase plasmogen activator (uPA), (2) plasminogen, (3) trypsin-1, (4) Nav1.5 (SCN5A), (5) 5-hydroxytryptamine receptor (5HT) isoforms 2A, 2C, 7, 1B, 2B, 1D, and 6, (6) dopamine receptor 2 (D2), (7) histamine 1 receptor (H1), (8) α-adrenergic receptors (1A, 1D, 2B, 2A, and 2C), and (9) Cav1.2 (CACNA1C).
  CEREP screening of this compound identified several of these predicted targets including: (1) 5HTs (1A, 1B, 2A, 2C, 3, 5a, 6, and 7), (2) Cav1.2 (CACNA1C), (3) D1, D2, D3, and D5, (4) Nav1.5 (SCN5A), (5) α-adrenergic receptors (1 and 2), (6) β-adrenergic receptors (1 and 2), and (7) H2. However, CEREP screening also identified the muscarinic receptors (1, 2, 3, 4, and 5) isoforms, opioid receptor like (ORL) and benzodiazepine receptor (BZD) as off-targets, which were not predicted by the OTSA process. It should be noted that OTSA predicted H1, but CEREP identified H2 and this OTSA prediction was not considered as positive prediction.
2. **A-777903**
  A-777903 is an oral compound that was designed to antagonize melanin-concentrating hormone-1 (MCH-1). A-777903 effectively binds with MCH-1 with an IC_50_ of 16 nM (Basso et al., 2006). The OTSA process identified MCH-1 as the top scoring target. Additional predicted targets included: (1) 5HTs (2,1A, 2B, 2C, and 7), (2) α -adrenergic receptor 1, (3) D1, 2 and 4, (4) δ, μ and σ-opioid receptors, (5) KCNH1 and 2, (6) κ -type opioid receptor, (7) muscarinic receptor 1, (8) Na^2+^ dependent serotonin transporter, (9) somatostatin type 3 receptor and (10) H1.
  The CEREP assay identified only a few of these predicted targets: (1) 5HTs isoforms and (2) D1 and 2. However, CEREP identified Cav1.2 and β adrenergic receptor as off-targets, which were not predicted by any of the methods within the OTSA. It should be noted that the CEREP assay captured interaction with H2, but that OTSA predicted H1 receptor as an off-target, indicating that the OTSA process may generally help in the identification of target families rather than specific isoforms, as noted with A-317567. Likewise, the OTSA predicted α-adrenergic receptor, which is structurally similar to the β isoform
3. **A-1411735**
  A-1411735 is a protein kinase C theta (PKCθ) inhibitor that was evaluated as a potential treatment for autoimmune diseases (George et al., 2014). A-1411735 inhibits PKCθ with an IC_50_ of 23 nM. This compound also showed inhibitory activities against other PKC isoforms such as α (1,920 nM), β (598 nM), δ (322 nM), ε (92 nM), and γ (16,698 nM). However, A-1411735 is highly selective across a panel of 81 kinases, displaying only weak inhibition (in μM range) with Cdc-like kinase 2 (CLK2), GSK3α, and cyclin dependent kinase 8 (CDK8). Additionally, profiling across the AbbVie 70 panel resulted in only two hits namely, Cav1.2 (CACNA1C) and κ opioid receptor (KOP). The OTSA process predicted all the PKC isoforms as potential targets including PKCθ with a score ranging from 0.7 to 1, as well as Cav1.2 (CACNA1C) with a score of 0.7. This chemotype was also predicted to bind to CDK5 and 8 with a low score <0.5, below the threshold pseudo-score that is used in this study. However, the 3Decision binding site similarity approach independently identified CDK9, a close homolog of CDK8, as a target with a binding site similarity of 63%. Nevertheless, the OTSA process did not predict the KOP as possible target in disagreement with *in vitro* data.
4. **A-908292 (S) and A-875400 (R)**
  A-908292 (S-enantiomer) is a small molecule inhibitor of acetyl CoA carboxylase 2 (ACC2) that was being developed to treat lipidemia (Waring et al., 2008). A-908292 selectively inhibits ACC2 and ACC1 with an IC_50_ value of 23 nM and 1.1 μM, respectively. On the other hand, A-875400 (R-enantiomer) inhibits ACC2 and ACC1 with an IC_50_ of 18 μM and 15 μM, respectively. Gene expression analysis of rat livers showed that both A-908292 and A-875400 were highly similar to a peroxisome proliferator-activated receptor (PPARα) activator (Waring et al., 2008). The OTSA process predicted three possible metabolites for both A-908292(S) and A-875400(R). One of these metabolites was predicted to interact with PPARα, prostaglandin D2 receptor 2, ACC1 and ACC2. The parents (i e. A-908292 and A-875400) were predicted to bind to ACC1 and 2, KCNQ2, glutaminyl-peptide cyclotransferase, Rho-associated, coiled-coil-containing protein kinase 1 (Rock1), voltage-gated potassium channel subunit K_v_7.4 (KV7.4), prostaglandin E_2_ receptor 4 (EP4), 5HT_2c_, CB1 and glutamate receptor 5 (mGlu5). However, an interaction was not predicted between the parents and PPARα indicating a possible functional role of the predicted metabolite. CEREP data were not generated for the predicted metabolites and hence these predicted interactions cannot be confirmed.
5. **A-803467**
  A-803467 is a potent and selective Na_v_ 1.8 sodium channel blocker. The measured IC_50_ with Na_v_ 1.8, Na_v_1.2, Nav1.3, Na_v_1.5, and Na_v_1.7 are 79 nM, 9.49, 11.7, 32.8, and 35 μM, respectively (Jarvis et al., 2007). CEREP screening showed significant binding of A-803467 with adenosine receptors (A3 and A2A), 5HT_2a_, CB1, and histamine receptor 2. OTSA identified all the isoforms of Na channels as well as 5HT_2a_. None of the OTSA methods predicted CB1, A3, A2A, and histamine receptors as possible targets. However, the OTSA predicted Substance P receptor, melatonin receptor and Bromodomain-containing protein 4 (BRD4) as high scoring off-targets, which were not part of CEREP panel.
  Taken together, the OTSA process correctly identified the pharmacological target as well as a significant number of off-targets confirmed *in vitro*.

### Physicochemical Properties

Several studies have demonstrated a relationship between molecular promiscuity (ability of molecules to bind and interact with multiple targets) and toxicity. A number of physicochemical properties have been suggested as contributing to higher promiscuity, such as lipophilicity (as calculated by clogP), high MW and the presence of ionizable amines (leading to high pKa) (Tarcsay and Keseru, 2013). For example: (1) Pfizer screened 75000 compounds against 220 assays and found lipophilicity (logP) to be positively correlated with promiscuity (Hopkins et al., 2006); (2) Pfizer also screened 245 preclinical compounds and found increased toxic outcomes for less polar compounds (i.e., lipophilic compounds) (Hughes et al., 2008); (3) AstraZeneca screened 2133 drug molecules along with reference compounds against 200 CEREP targets and found significant correlation between promiscuity and logP, MW, and pKa values (Yang et al., 2010); (4) Roche screened 2413 compounds against 141 safety associated targets and suggested that promiscuity was connected with logP and pKa (Peters et al., 2012); and (5) Novartis queried 656 drugs in 73 assays, and logP and pKa were connected to promiscuity (Lounkine et al., 2012). Overall, these studies agree that lipophilicity (logP), MW, and pKa are key physicochemical factors that play an important role in drug promiscuity.

Consequently, several of these physicochemical properties were calculated for the three categories of compounds (approved, discontinued, and internal compounds) evaluated in the OTSA in order to understand any potential bias related to differences in physicochemical properties between the three categories of compounds, but also in order to evaluate whether the number of predicted off-targets would be associated with any specific physicochemical feature.

Several computed physiochemical properties of the 401 approved drugs were statistically different from those of the 456 discontinued drugs. For example, lipophilicity (logP, clogD), MW, and pKa were statistically different between the approved and discontinued drugs, suggesting that these physicochemical properties may have a role, at least in part, in the discontinuation of the drugs. However, the physicochemical properties of the internal compounds were mostly similar to the approved drugs, except for lipophilicity. Moreover, both approved and discontinued drugs were predicted to bind with more off-targets with MW (300–500)/TPSA (>200) and less off-targets with MW (<200 or >700), suggesting a range of MW and TPSA more prone to promiscuity. Nevertheless, no other single or combinations of physicochemical properties correlated with the number of predicted off-targets for these drug categories, suggesting that physicochemical properties alone are insufficient to understand promiscuity and off-targets for small molecules.

### Limitations of Chemocentric Off-Target Prediction Methods

In spite of their unique advantages, the ligand-centric (chemical similarity-based) methods within the OTSA are inherently biased by the chemical coverage offered by the active molecules present in those published sources (patents and publications), which significantly limits the capacity of prediction for novel molecules not represented within the training set, as evident from the results for the 15 internal compounds. For example, when evaluating internal compounds, the OTSA process failed to predict some experimentally observed interactions, as these interactions were new to the OTSA framework (i.e., not covered by the databases). For example, A-803467 displayed significant *in vitro* activity with adenosine receptors (A1, A2A), BZD, and opioid μ receptor. None of the OTSA methods predicted these as possible off-targets. In particular, BZD interactions were not predicted by the OTSA process not only for A-803467 but also for A-922500, as the reference database is not enriched with this pattern of interactions. This indicates the need to complement the training set with internal compounds that cover the institutional chemical space. Another major disadvantage of ligand-centric similarity-based approaches is that inactive, but structurally similar compounds can generate false positive predictions. For example, some predicted interactions for the 15 internal compounds were not confirmed *in vitro*. A-993610 was predicted to bind to the μ opioid receptor, CB1 and H3. However, none of these were identified as hit by *in vitro* CEREP screening. However, since the OTSA approach uses not only chemical similarity, but also pharmacophoric features as an add-on query (such as the inclusion of positive, negative, hydrophobic, hydrogen bond donor and acceptors features), the number of false positive interactions is limited. Generally, pharmacophoric signatures consist of target recognition elements held in geometrically well-defined positions within a small molecule. Typically, five recognition elements are commonly recognized by the binding site of any biological targets: hydrophobe-aromatic elements, hydrogen bond acceptors and donors, and negatively and positively charged groups, and those are used as the pharmacophoric features. Complementarity between pharmacophoric signatures of ligand and target determines their intermolecular recognition (Liu et al., 2010).

False positive interactions are also expected, because not all active chemotype interactions with particular targets have been curated, as state above. Furthermore, the prediction for targets with limited bioactive/inactive ligands is not possible by simple cheminformatics approaches. For this reason, we have been regularly integrating different SAR databases with new chemistry and computational methodologies to minimize the rate of false negative predictions. Nevertheless, this clearly indicates a need to further complement the database with additional chemotypes and experimentally generated interactions. Finally, as mentioned earlier, the OTSA approach can only predict whether a compound can bind to a target or not and this may not reflect a functional interaction. Likewise, all the existing 2-D similarity methods do not distinguish between enantiomers. This was illustrated with A-875400, the R-enantiomer of A-908292, which is significantly weaker against ACC2, but for which scores similar to A-908292 were generated. Likewise, data from the internal compounds suggest that distinction of selectivity against various isoforms of the same target family may be limited with the OTSA approach, as illustrated with A-1411735 and A-903482.

### Limitations of Protein Structure-Based Target Prediction Methods

Even when significantly different in amino acid sequence and with limited structural homologies, proteins may share binding site similarity, enabling them to bind similar ligands (Haupt et al., 2013). This aspect is the rationale behind target-centric prediction methods, which can be used to identify off-targets for novel chemistries (i.e., chemistry with no SAR data). With these methods, small molecule ligand libraries are queried against the three-dimensional pockets of the human structural proteome for spatial and electronic fitness. The complementarity between the protein drug pockets and chemical structure is computed using automated docking tools such as Glide and AutoDock (Friesner et al., 2004; Kellenberger et al., 2004; Trott and Olson, 2010). In addition, the binding site similarity index is used to identify off-targets. It is based on the assumption that structurally similar binding sites have similar molecular function and thus, are likely to bind to structurally similar compounds.

The OTSA target-centric approach uses >140,000 high-resolution X-ray structures from the Protein Databank (PDB) and an internal structural database. Unlike the ligand-centric methods, this method can be used only when either the experimental or homology model of the protein structure complexed with a compound of interest (i.e., compound for which off-target prediction is needed) is available. This limits the utility of target structure-based off-target prediction. In spite of the availability of a large number of 3-D approaches for the comparison of protein-binding sites, the number of off-target predictions is generally low, as these methods do not explicitly consider the binding pocket dynamics, water mediated effects, or importance of the interactions with co-factors as part of binding site description query. Moreover, the 3-D binding site search of our OTSA is low-throughput and can only be used for a limited number of key preclinical and clinical compounds. The physiological target for A-1411735 and A-1390577 is PKCθ. Interestingly, this 3-D binding site search identified CDK isoforms and GSKβ (not GSKα) binding sites features are 76 and 60% similar, respectively, to the 3-D binding site of PKCθ, in partial agreement with *in vitro* studies. This implicates that the 3-D based method not only identifies pharmacological targets correctly, but also supplements additional off-targets which are not identified by chemo-informatics methods.

### Strengths and Limitations of the OTSA

A reasonably performant computational method(s) (either 2-D- or protein structure-based) should ideally identify the “true-hits” (i.e., pharmacological target and other confirmed CEREP targets) as the top-scoring targets for the compounds tested. The ligand-centric methods of the OTSA predicted most of the experimentally confirmed interactions for the discontinued and approved drugs as the top scoring targets, suggesting that OTSA has an appropriate mix of 2-D methods and databases for target prediction. In particular, in this work, chemo-centric methods alone captured a total of 1,898 (~4.1 interactions/compound) confirmed off-target interactions for the 456 discontinued drugs. These interactions were mainly from 886 distinct targets. Of these, 600 targets were also identified as off-targets for approved drugs. Thus, 286 off-targets distinguished the approved from the discontinued drugs. Among these, 146 (51%) targets have been previously confirmed *in vitro*. The remaining 140 targets (49%) were new and therefore not confirmed, such that the exact performance characteristics of our computational approach could not be fully calculated. Nevertheless, the OTSA process uniquely differentiated approved drugs from the discontinued drugs with the identification of interactions of latter with cytochrome p450 (CYP) isoforms, T, N, and L type calcium channel voltage-dependent channels, sodium channels subunit α isoforms (1–9), cyclin-dependent kinase (CDK) isoforms (2, 5, 6, 8, 9), Phosphodiesterase 3 (PDE3), and hERG K^+^ channel, suggesting dialing-out interactions with these targets might significantly enhance the chance of success in preclinical studies. Overall, 56% of the predicted interactions for the 15 internal compounds were in agreement with the CEREP data, suggesting that the performance of this computational tool is still modest. The most frequent *in vitro* identified off-targets for these compounds were 5HTs (1a, 1b, 2a, 2b, 2c, 3, 5a, and 7), BZD, CB1, Cav1.5 Nav1.2, Cl^−^ (GABA-gated) channels, and D1, D2, and D4. Particularly, of the 15 compounds, 5 compounds interacted with Nav1.2, 4 interacted with Cav1.5 receptors and BZD and 3 compounds showed activity against 5HT_2a_ and 5HT_2c_, in reasonable agreement with *in vitro* studies. Since the reference database does not have BZD interacting compounds, none of the OTSA methods predicted interactions with BZD, further emphasizing the need for expansion of the reference chemistry database. In another example, the OTSA process predicted GSK3β, the tyrosine kinase Fyn, CDK8, Nuclear receptor subfamily 4 group A member 2 (NR4A2), and 5HT_2b_ as high-scoring off-targets for A-277249, in excellent agreement with internal *in vitro* profiling data that showed significant inhibition of GSK3β and CDK8 (0.4 and 0.2 μM, respectively). Moreover, transcriptomics profiling of this compound revealed that the observed hepatotoxicity of A-277249 was mediated through the aryl hydrocarbon nuclear receptor (AhR) pathway (Waring et al., 2002). Interestingly, NR4A2 is one of the key target genes for the AhR transcription factor, suggesting a potential role of OTSA in the identification of off-targets which may complement transcriptomics data.

The *in vitro* binding data alone are insufficient to assess preclinical and clinical risks, and the prediction of an off-target interaction does not necessarily translate to an effect. Various factors, such as efficacious systemic exposure levels, volume of distribution, tissue expression and off-target residence time must be taken into consideration while assessing the potential risk linked to these predicted off-targets. Recently, 19 clinically well-characterized small molecule kinase inhibitors were screened against three cardiovascular (CV) relevant kinases [Ribosomal Protein S6 Kinase B1(RPS6KB1), Focal adhesion kinase (FAK) and Serine/Threonine Kinase 35 (STK35)] at four concentrations centered around their respective clinical human C_max_ concentration value (Lamore et al., 2017). The IC_50_ of each compound in relation to C_max_ was used to differentiate CV toxic and non-toxic compounds. In this model, if the measured IC_50_ value against these kinases was less than the therapeutic C_max_ concentration, then the compound was predicted to be cardiotoxic. On the other hand, if the IC_50_ value against these kinases was greater than the therapeutic C_max_ concentration, the compounds were predicted to be “cardiac safe.” Interestingly, this simple mathematical model connecting therapeutic C_max_ concentrations and IC_50_ values against only three cardiac relevant kinases correctly predicted 3 of 5 “cardiac safe” compounds and 12 of 15 cardiotoxic compounds, illustrating the importance of including *in vivo* exposure levels in the assessment of off-target mediated toxicities. Additionally, the critical factor for sustained drug efficacy (on target binding) and toxicity (off-target binding) is not the binding affinity, but the residence time of the drug molecule within the binding pocket of on/off-targets. Several examples confirm the role of residence time in prolonged target-mediated *in vivo* outcomes (Copeland et al., 2006). The need to integrate these data with off-target interaction information represents the future of safety screening and mechanism identification investigations, and we are progressively incorporating this type of information in our assessments during lead optimization programs.

During the lead identification stage, large compound libraries (usually>100,000) are generally screened against the target of interest to discover novel lead molecules. It is not possible to work on the chemical optimization of each and every lead identified. Therefore, at this stage, the inclusion of key physicochemical property filters (such as clogP, TPSA, pKa, MW, number of rotatable bonds, number hydrogen bond donor, and acceptors) can bias the odds in favor of finding successful leads. The current work suggests the physicochemical properties of approved drugs (MW, logP, pKa, and logS) are statistically different from those of discontinued drugs, in agreement with other published reports (Wenlock et al., 2003; Shultz, 2018). Additionally, the predicted adverse off-target interaction information might help in selecting the right leads for further advancement, especially when considering moving to *in vivo* preclinical studies when resources and compound requirements are high. Finally, OTSA is not intended to replace *in vitro* off-target binding and toxicity testing. Rather, it should serve as a filter to increase the probability of success and guide the optimization of compounds toward a chemical space with lower safety liabilities. Furthermore, this computational tool can provide mechanistic clarity through a better understanding of polypharmacology, and by providing testable target-mediated toxicity hypotheses for toxic changes observed in *in vivo* studies. Robustly predicted off-interactions typically lead to orthogonal confirmatory studies to test these predictions. The OTSA model should then be refined by incorporating new chemical matter and experimental data in order to further improve the performance characteristics and utility of the off-target predictions.

## Conclusions

In summary, a novel computational framework, termed OTSA, was developed to predict off-target interactions and potential associated toxicities for small molecules. OTSA was tested using publicly available and internal compounds, and results suggest that this computational approach can provide value when appropriately used and when its limitations are properly understood. In particular, this computational tool should enable improving the selectivity of novel drug candidates by limiting undesirable off-target interactions and by decreasing non-clinical safety-related attrition. The observation of >49% (56, 49.5, and 78% for internal compounds, discontinued and approved drugs, respectively) predictive value for confirmed interactions in each compound set is promising considering that (1) our OTSA process includes metabolites in the prediction, which are not assessed by CEREP (there may be additional targets confirmed if metabolites were assessed) and (2) not all of the predicted interactions were included in the CEREP screens and, as such, may also be accurate predictions. Finally, this study indicated that the computed lipophilicity (clogP), charge (pKa) and MW of the discontinued drugs are generally in higher range compared to the approved drugs. This work also suggests that the effective use of these computed simple physiochemical properties together with the OTSA predicted adverse interactions may form an integrated framework that can be used in lead optimization and preclinical studies.

## Author Contributions

All authors are aware of the manuscript and have contributed significantly to its completion. In addition, all authors are employed at AbbVie and the appropriate disclosures are included on the title page along with keywords.

### Conflict of Interest Statement

All authors are employed by AbbVie. The design, study conduct, and financial support for this research was provided by AbbVie. AbbVie participated in the interpretation of data, review, and approval of the publication.

## Supplementary Material

Supplementary Table S1 Summary of contributions from different 2-D target prediction methods for discontinued and approved drugs.

Supplementary Data Sheet S1 Statistical analysis on the computed physiochemical properties of the approved and discontinued drugs.

Supplementary Data Sheet S2 Summary of computationally predicted interactions for the **(A)** approved and **(B)** discontinued drugs.

Supplementary Data Sheet S3 Violin plots showing distributions of **(A)** MW, **(B)** pKa, **(C)** clogD, **(D)** logS, **(E)** MDCK (nm/sec), **(F)** clogP, **(G)** TPSA, **(H)** sp^3^ count, **(I)** number of rotatable bonds, and **(J)** Caco_2_ permeability (nm/sec) for approved (left) and discontinued (middle) drugs and AbbVie's compounds (right). The 75th and 25th percentile, median, and 95% confidence interval and standard deviation (SD) are shown.
