# Supplementary material for: Novel Computational Approach to Predict Off-Target Interactions for Small Molecules
Source: Front Big Data. 2019 Jul 17;2:25. doi: 10.3389/fdata.2019.00025 (PMC7931946; doi:10.3389/fdata.2019.00025)
Supplement: Supplementary Table S1 — Summary of contributions from different 2-D target prediction methods for discontinued and approved drugs. [file Table_1.docx]

**Supplemental Table 1**

**Summary of contributions from different 2-D target prediction methods for discontinued and approved drugs.**

| **Number of Methods** | **Discontinued – Predicted interaction** | **Approved – Predicted interactions** |
| --- | --- | --- |
| 6 | 706 | 923 |
| 5 | 940 | 862 |
| 4 | 866 | 598 |
| 3 | 1411 | 577 |
| Training set | 504 | 1107 |
