# Supplementary figures and images for: Novel Computational Approach to Predict Off-Target Interactions for Small Molecules
Source: Front Big Data. 2019 Jul 17;2:25. doi: 10.3389/fdata.2019.00025 (PMC7931946; doi:10.3389/fdata.2019.00025)

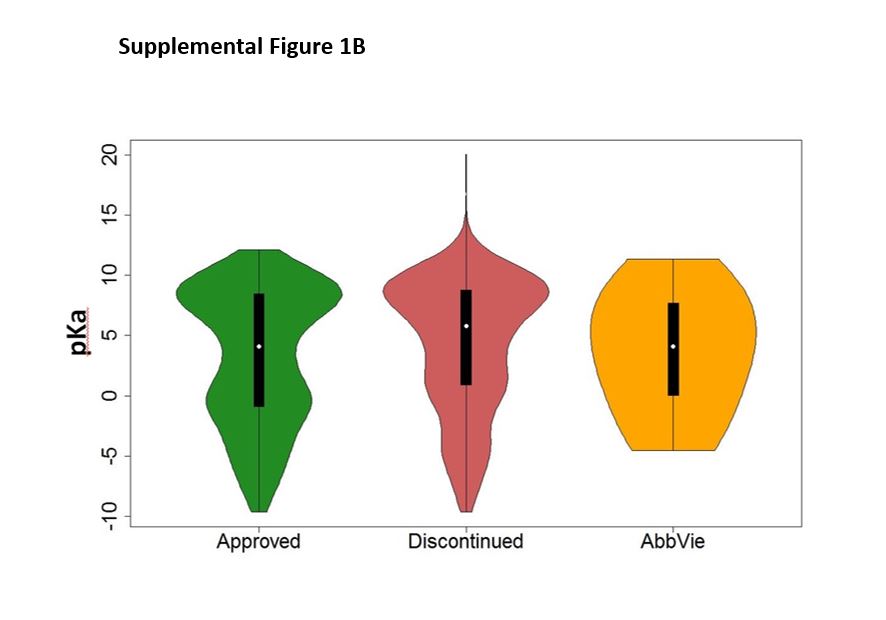

Supplement: Supplementary Data Sheet S3 — Violin plots showing distributions of (A) MW, (B) pKa, (C) clogD, (D) logS, (E) MDCK (nm/sec), (F) clogP, (G) TPSA, (H) sp3 count, (I) number of rotatable bonds, and (J) Caco2 permeability (nm/sec) for approved (left) and discontinued (middle) drugs and AbbVie's compounds (right). The 75th and 25th percentile, median, and 95% confidence interval and standard deviation (SD) are shown. [file Data_Sheet_3.zip › Supplemental_Fig1b.JPG]

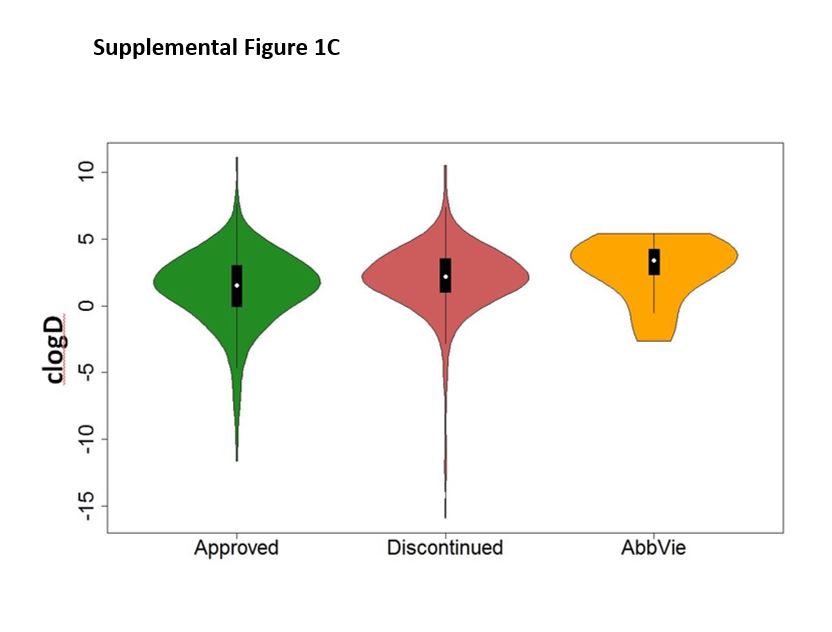

Supplement: Supplementary Data Sheet S3 — Violin plots showing distributions of (A) MW, (B) pKa, (C) clogD, (D) logS, (E) MDCK (nm/sec), (F) clogP, (G) TPSA, (H) sp3 count, (I) number of rotatable bonds, and (J) Caco2 permeability (nm/sec) for approved (left) and discontinued (middle) drugs and AbbVie's compounds (right). The 75th and 25th percentile, median, and 95% confidence interval and standard deviation (SD) are shown. [file Data_Sheet_3.zip › Supplemental_Fig1c.JPG]

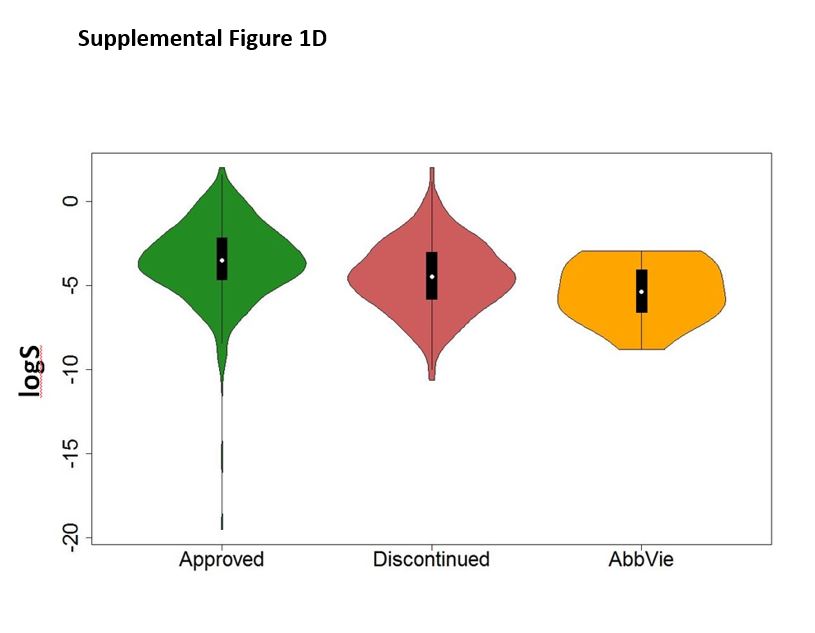

Supplement: Supplementary Data Sheet S3 — Violin plots showing distributions of (A) MW, (B) pKa, (C) clogD, (D) logS, (E) MDCK (nm/sec), (F) clogP, (G) TPSA, (H) sp3 count, (I) number of rotatable bonds, and (J) Caco2 permeability (nm/sec) for approved (left) and discontinued (middle) drugs and AbbVie's compounds (right). The 75th and 25th percentile, median, and 95% confidence interval and standard deviation (SD) are shown. [file Data_Sheet_3.zip › Supplemental_Fig1d.JPG]

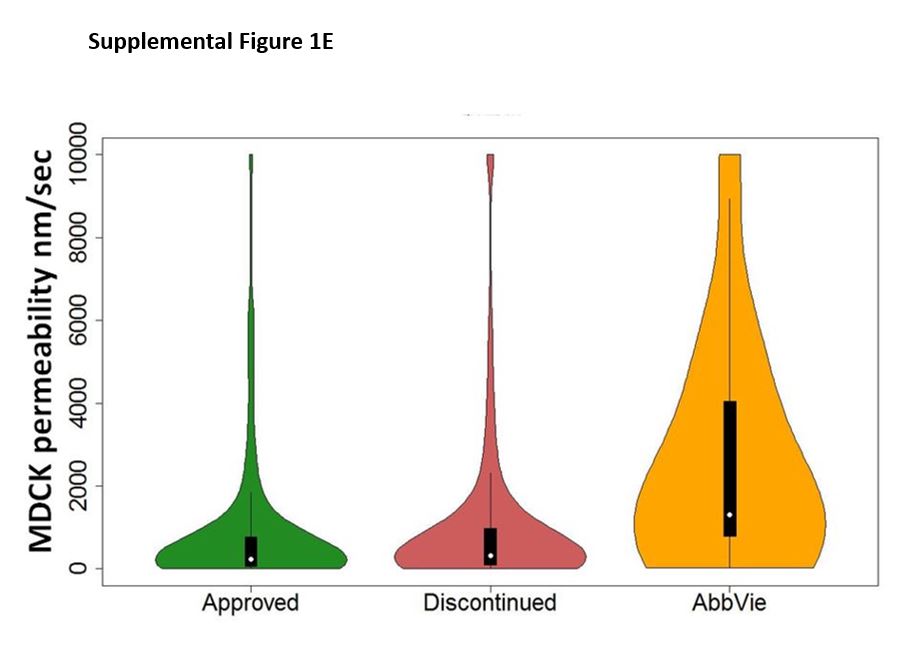

Supplement: Supplementary Data Sheet S3 — Violin plots showing distributions of (A) MW, (B) pKa, (C) clogD, (D) logS, (E) MDCK (nm/sec), (F) clogP, (G) TPSA, (H) sp3 count, (I) number of rotatable bonds, and (J) Caco2 permeability (nm/sec) for approved (left) and discontinued (middle) drugs and AbbVie's compounds (right). The 75th and 25th percentile, median, and 95% confidence interval and standard deviation (SD) are shown. [file Data_Sheet_3.zip › Supplemental_Fig1e.JPG]

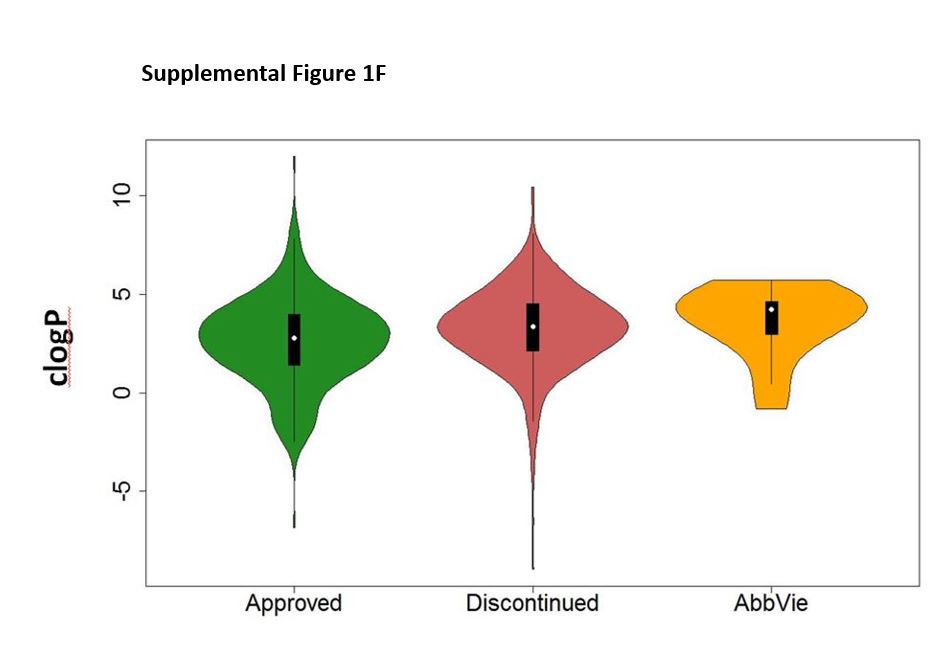

Supplement: Supplementary Data Sheet S3 — Violin plots showing distributions of (A) MW, (B) pKa, (C) clogD, (D) logS, (E) MDCK (nm/sec), (F) clogP, (G) TPSA, (H) sp3 count, (I) number of rotatable bonds, and (J) Caco2 permeability (nm/sec) for approved (left) and discontinued (middle) drugs and AbbVie's compounds (right). The 75th and 25th percentile, median, and 95% confidence interval and standard deviation (SD) are shown. [file Data_Sheet_3.zip › Supplemental_Fig1f.JPG]

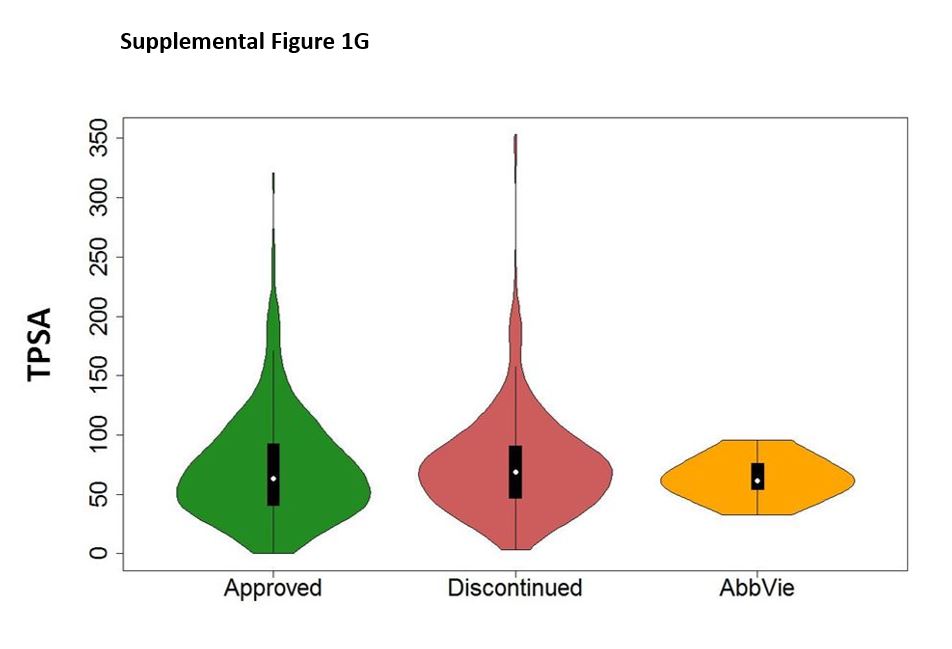

Supplement: Supplementary Data Sheet S3 — Violin plots showing distributions of (A) MW, (B) pKa, (C) clogD, (D) logS, (E) MDCK (nm/sec), (F) clogP, (G) TPSA, (H) sp3 count, (I) number of rotatable bonds, and (J) Caco2 permeability (nm/sec) for approved (left) and discontinued (middle) drugs and AbbVie's compounds (right). The 75th and 25th percentile, median, and 95% confidence interval and standard deviation (SD) are shown. [file Data_Sheet_3.zip › Supplemental_Fig1g.JPG]

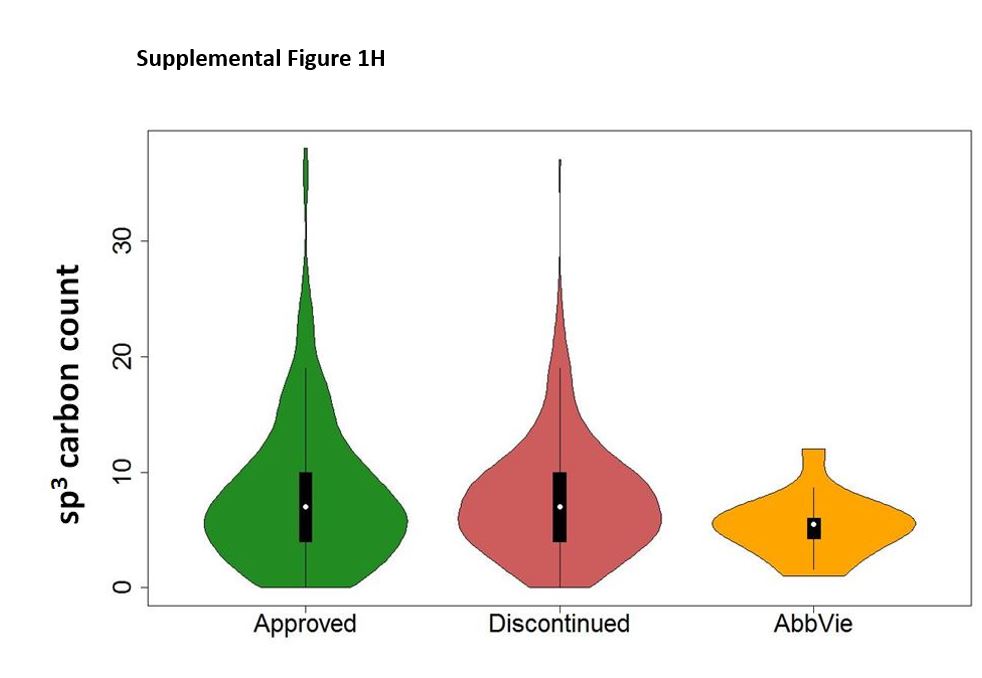

Supplement: Supplementary Data Sheet S3 — Violin plots showing distributions of (A) MW, (B) pKa, (C) clogD, (D) logS, (E) MDCK (nm/sec), (F) clogP, (G) TPSA, (H) sp3 count, (I) number of rotatable bonds, and (J) Caco2 permeability (nm/sec) for approved (left) and discontinued (middle) drugs and AbbVie's compounds (right). The 75th and 25th percentile, median, and 95% confidence interval and standard deviation (SD) are shown. [file Data_Sheet_3.zip › Supplemental_Fig1h.JPG]

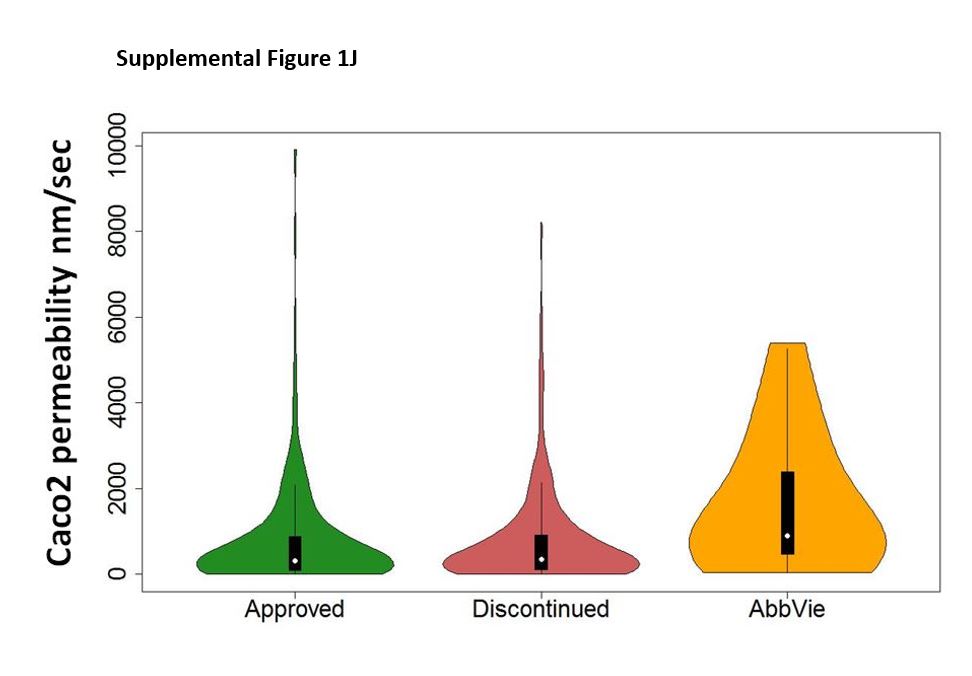

Supplement: Supplementary Data Sheet S3 — Violin plots showing distributions of (A) MW, (B) pKa, (C) clogD, (D) logS, (E) MDCK (nm/sec), (F) clogP, (G) TPSA, (H) sp3 count, (I) number of rotatable bonds, and (J) Caco2 permeability (nm/sec) for approved (left) and discontinued (middle) drugs and AbbVie's compounds (right). The 75th and 25th percentile, median, and 95% confidence interval and standard deviation (SD) are shown. [file Data_Sheet_3.zip › Supplemental_Fig1j.JPG]

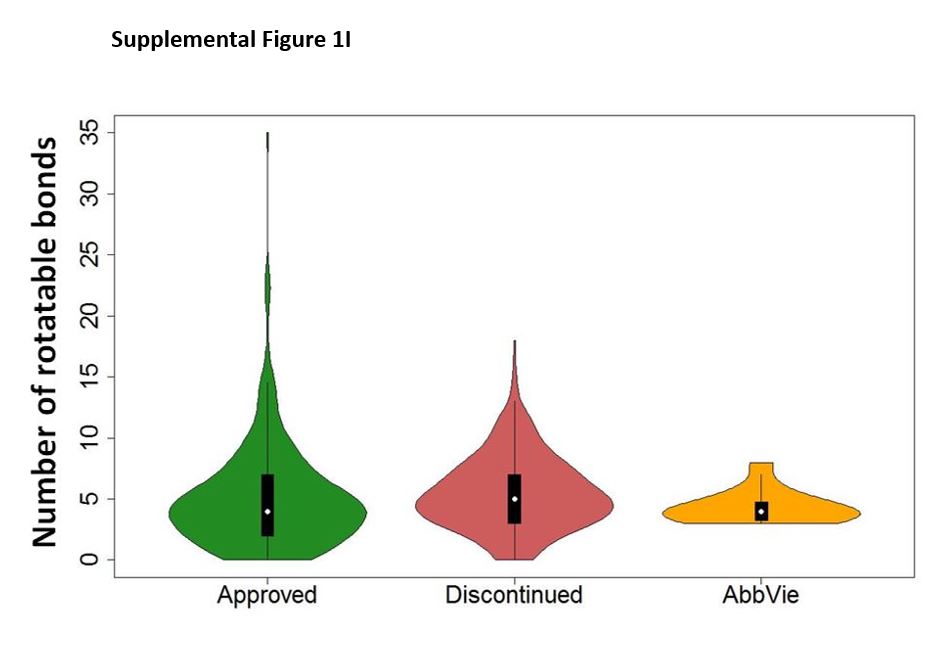

Supplement: Supplementary Data Sheet S3 — Violin plots showing distributions of (A) MW, (B) pKa, (C) clogD, (D) logS, (E) MDCK (nm/sec), (F) clogP, (G) TPSA, (H) sp3 count, (I) number of rotatable bonds, and (J) Caco2 permeability (nm/sec) for approved (left) and discontinued (middle) drugs and AbbVie's compounds (right). The 75th and 25th percentile, median, and 95% confidence interval and standard deviation (SD) are shown. [file Data_Sheet_3.zip › Supplemenal_Fig1i.JPG]

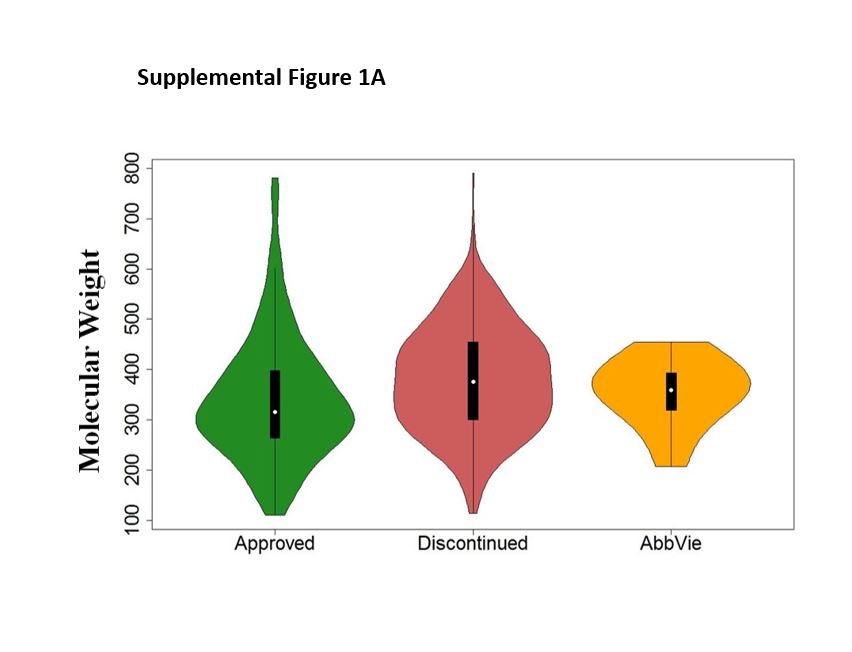

Supplement: Supplementary Data Sheet S3 — Violin plots showing distributions of (A) MW, (B) pKa, (C) clogD, (D) logS, (E) MDCK (nm/sec), (F) clogP, (G) TPSA, (H) sp3 count, (I) number of rotatable bonds, and (J) Caco2 permeability (nm/sec) for approved (left) and discontinued (middle) drugs and AbbVie's compounds (right). The 75th and 25th percentile, median, and 95% confidence interval and standard deviation (SD) are shown. [file Data_Sheet_3.zip › Supplemental_Fig1a.jpg]
